# Pesticide Exposure and Gynecological Cancers: A Review of Epidemiological Evidence and Mechanistic Pathways

**DOI:** 10.3390/diseases14080273

**Published:** 2026-07-29

**Authors:** Maedeh Mirasheh, Zahra Shahabinia, Afrooz Mazidimoradi, Toktam Soleimani, Leila Allahqoli, Hamid Salehiniya

**Affiliations:** 1 Department of Midwifery and Reproductive Health, School of Nursing and Midwifery, Tehran University of Medical Sciences, Tehran 1996713883, Iran; 2 Geriatric Health Research Center, Birjand University of Medical Sciences, Birjand 9717853577, Iran; 3 Health Assistant Department, Shiraz University of Medical Sciences, Shiraz 7193711351, Iran; 4 Emergency Department, 9 Dey Educational Hospital, Torbat Heydariyeh Univercity of Medical Sciences, Torbat Heydariyeh 9518831166, Iran; 5 Faculty of Health Sciences, Cyprus International University, Nicosia 99258, Cyprus; 6 Department of Epidemiology and Biostatistics, School of Health, Social Determinants of Health Research Center, Birjand University of Medical Sciences, Birjand 9717853577, Iran

**Keywords:** pesticides, gynecological cancers, ovarian cancer, endometrial cancer, cervical cancer, endocrine disruption, oxidative stress

## Abstract

Environmental exposure to pesticides is considered a risk factor for gynecological cancers, contributing to their onset and progression through various mechanisms. This narrative review investigates the role of different pesticides in the development and progression of ovarian, endometrial, and cervical cancers, and provides a comprehensive synthesis of epidemiological findings along with molecular mechanisms, highlighting carcinogenic pathways, conflicting findings, and potential therapeutic implications. Relevant epidemiological and experimental studies published between 2000 and 2025 were identified through searches of major scientific databases. Various pesticides, particularly organochlorines, contribute to gynecological cancers through mechanisms such as estrogen mimicry, DNA damage, oxidative stress, increased pro-inflammatory cytokines, and disruption of cellular signaling pathways. However, most studies found no significant association between certain pesticides, such as Triazines and Atrazine, and gynecological cancers. Furthermore, some pesticides, like Carbendazim, exhibit a dual role; while carcinogenic, they can also serve therapeutic purposes in cancer treatment when utilized with nanotechnology. Overall, pesticide exposure is a significant factor in the development and progression of women’s cancers, although the strength of the evidence varies across pesticide classes. Despite numerous studies, contradictory findings necessitate further research to clarify causal relationships.

## 1. Introduction

Gynecological cancers, including cervical, ovarian, uterine, vaginal, and vulvar cancers, represent a serious threat to women’s health [1]. Cervical, ovarian, and endometrial cancers are the most prevalent. According to data from the International Agency for Research on Cancer (IARC), approximately 1.33 million new cases of gynecological cancers occur annually, accounting for 540,000 deaths in 2022. Cervical cancer ranks as the fourth most common cancer in women globally, with an estimated 660,000 new cases and 350,000 deaths per year [2,3]. In high-income countries, endometrial cancer is the most common gynecological malignancy, with incidence rates rising steadily [4]. Ovarian cancer is the eighth most common cancer in women, responsible for 4.7% of all cancer-related deaths in 2020 [5,6,7]. Various risk factors contribute to ovarian, endometrial, and cervical cancers, including environmental exposure to pesticides [8,9,10]. It should be noted that the main cause of cervical cancer is human papillomavirus (HPV) [11]. Environmental factors, including pesticide exposure, may play a role as potential contributors by causing oxidative stress, endocrine disruption, or other mechanisms in the development of cervical cancer [12,13,14,15,16].

Pesticides encompass a broad range of herbicides, fungicides, and insecticides [17]. They can exert adverse effects on human health through occupational exposure (e.g., agriculture), dietary exposure to pesticide residues present in food and drinking water, residential exposure resulting from living near agricultural areas where pesticides are applied, or indirectly via spousal exposure [18,19,20,21]. These chemicals may contribute to the development of gynecological cancers by increasing pro-inflammatory cytokines, inducing oxidative stress, and disrupting cancer-related signaling pathways [12,13,14,15,16].

A shared mechanism in the etiology of ovarian, endometrial, and cervical cancers involves endocrine disruption and the estrogen-mimicking effects of certain pesticides [16,22,23]. Pesticides such as organochlorines, atrazine, chlorpyrifos, and fenitrothion possess estrogenic properties that disrupt endocrine function, thereby playing a role in gynecological cancer pathogenesis [22]. Similarly, the fungicide cyprodinil has been implicated in endometrial cancer [23], and organochlorines have been linked to cervical cancer [16] through comparable mechanisms. Evidence from most reviewed studies supports a positive association between pesticide exposure and gynecological cancers [8,9,12,13,24,25,26,27,28,29]. However, contradictions exist in some findings [24,27,30,31,32,33]. This review examines the role of various pesticides and their associated mechanisms in the development and progression of ovarian, endometrial, and cervical cancers.

## 2. Methods

This study was conducted as a narrative review to synthesize and interpret existing epidemiological and mechanistic evidence regarding the association between pesticide exposure and gynecological cancers, specifically ovarian, endometrial, and cervical cancers. Unlike systematic reviews that focus on counting studies, this narrative approach emphasizes identifying common patterns, addressing contradictions, and providing a deeper understanding of biological pathways.

A comprehensive literature search was performed across major electronic databases, including PubMed, Scopus, and Web of Science. The search period covered publications from January 2000 to February 2025 to ensure the inclusion of recent advancements in molecular mechanisms and epidemiological data. The search strategy utilized a combination of keywords related to pesticides (e.g., Organochlorines, Triazines, Carbamates, Fungicides) and gynecological malignancies (Ovarian Cancer, Endometrial Cancer, Cervical Cancer), along with mechanistic concepts such as Endocrine Disruption, Oxidative Stress, and Epigenetic Changes. Additionally, reference lists of relevant review articles and key primary studies were manually screened to ensure comprehensive coverage of the topic.

Studies were included if they provided human evidence (cohort, case–control, cross-sectional) or experimental evidence (in vitro and in vivo) investigating the effects of pesticides on gynecological tissues. Key inclusion criteria involved clear definitions of pesticide types, specific reporting of cancer outcomes or associated mechanistic pathways. Data extracted from each study included study characteristics, pesticide class, route of exposure, statistical results (for epidemiological studies), and mechanistic findings (such as gene expression changes, cytokine levels, and DNA damage). Given the heterogeneity in study designs and outcome measures, a meta-analysis was not feasible. Therefore, the findings were synthesized using a narrative synthesis approach.

The results were organized by cancer type (ovarian, endometrial, cervical) and further categorized by pesticide class and shared biological mechanisms (e.g., endocrine disruption, oxidative stress, epigenetic modifications). The primary objective was to explain how these factors interact in cancer pathogenesis and to discuss observed inconsistencies across different studies. This approach allowed for a more holistic examination of the complex relationships between environmental exposure and carcinogenesis than quantitative methods alone could provide.

## 3. Results

The literature search initially identified 215 potentially relevant articles. After screening titles, abstracts, and full text based on predefined eligibility criteria, 30 studies spanning from 2000 to 2025 met the inclusion criteria and were included in this narrative review. The review encompasses case–control studies [8,12,24,26,27], prospective and retrospective longitudinal studies [25,30,34,35], and cross-sectional studies [8,29]. These epidemiological findings were supported by experimental and laboratory studies investigating the mechanistic effects of pesticides on carcinogenesis [12,14,22,36,,37,38,39,40].

As presented in Table 1, evidences linking pesticide exposure to gynecological cancers is supported by measurements of pesticide levels in blood and tissue samples [8,12,13,24,27,29] and genetic polymorphisms [24]. Key mechanisms identified include endocrine disruption and estrogenic effects [14,15,16,22,23,,37,41], alterations in cancer-related signaling pathways [13,15,16,31,37], oxidative stress [12], epigenetic changes [22], and altered expression of cell cycle-related genes [37,38].

The outcomes assessed included the role of various pesticides in the onset and progression of ovarian, endometrial, and cervical cancers.

### 3.1. Ovarian Cancer

#### 3.1.1. Association Between Pesticides and Ovarian Cancer Incidence

Ovarian cancer is one of the deadliest gynecological malignancies. In the United States, it is estimated that there will be 20,890 new cases and 12,730 deaths in 2025 [42]. Environmental exposure to pesticides has been linked to ovarian disorders, including cancer [9]. An ecological study in Brazil, which used total pesticide sales (in tons), also reported a significant positive association between pesticide exposure and ovarian cancer mortality [25] (Table 2).

##### Organochlorines

Organochlorine pesticides (OCPs) are associated with an increased risk of ovarian cancer [9]. For instance, in India, higher serum levels of β-HCH, endosulfan 1, endosulfan 2, p,p′-DDT, p,p′-DDE, and heptachlor, along with the simultaneous deletion of Glutathione S-transferase Mu 1 (*GSTM1*) and (Glutathione S-transferase Theta 1) *GSTT1* genes, were significantly higher in ovarian cancer patients compared to controls. These factors increase the risk of epithelial ovarian cancer (EOC) by elevating CA-125 levels [24]. Other studies in India reported significantly higher levels of OCPs, including α-, β-, and γ-hexachlorocyclohexane (α-HCH, β-HCH, γ-HCH), dieldrin, heptachlor, p,p′-DDT, and p,p′-DDE, in reproductive cancer patients, including ovarian cancer, compared to controls. Notably, older age was associated with lower serum OCP concentrations [8,12]. Similarly, total OCP levels (α-HCH, β-HCH, γ-HCH, heptachlor, aldrin, DDE, DDD, p,p′-DDT, and Σ-DDT) were significantly higher in ovarian cancer patients, with the highest burden observed in the 21–30 age group [8]. In EOC patients in India, tissue levels of β-HCH, heptachlor, heptachlor epoxide B (HTEB), p,p′-DDE, and endosulfan I were significantly elevated [13].

##### Triazines

Conversely, most studies in the USA and France found no significant association between exposure to triazines and atrazine and increased ovarian cancer risk [32,35]. While working in pig, fruit, or potato farms during puberty increased ovarian cancer risk, birth on such farms had a protective effect [35]. A 19.7-year longitudinal study examining public water atrazine levels and postmenopausal ovarian cancer risk initially showed higher atrazine levels in cases but lacked statistical significance. After adjusting for nitrate (NO3-N) and trihalomethanes (TTHM) levels, no significant association remained [30,34]. Consistent with this, a study in Kentucky found that individual atrazine measurements in public drinking water exceeded the permissible limit (3 µg/L) in 10 of 15 regions studied, and Atrazine exposure was inversely associated with ovarian cancer [33]. Laboratory studies in mice also indicated that adding atrazine to 7,12-dimethylbenz(a)anthracene (DMBA) reduced ovarian adenocarcinoma incidence, though not significantly [36].

#### 3.1.2. Mechanisms of Pesticide Action in Ovarian Cancer

##### Oxidative Stress and Inflammatory Pathways

**Figure 1:**
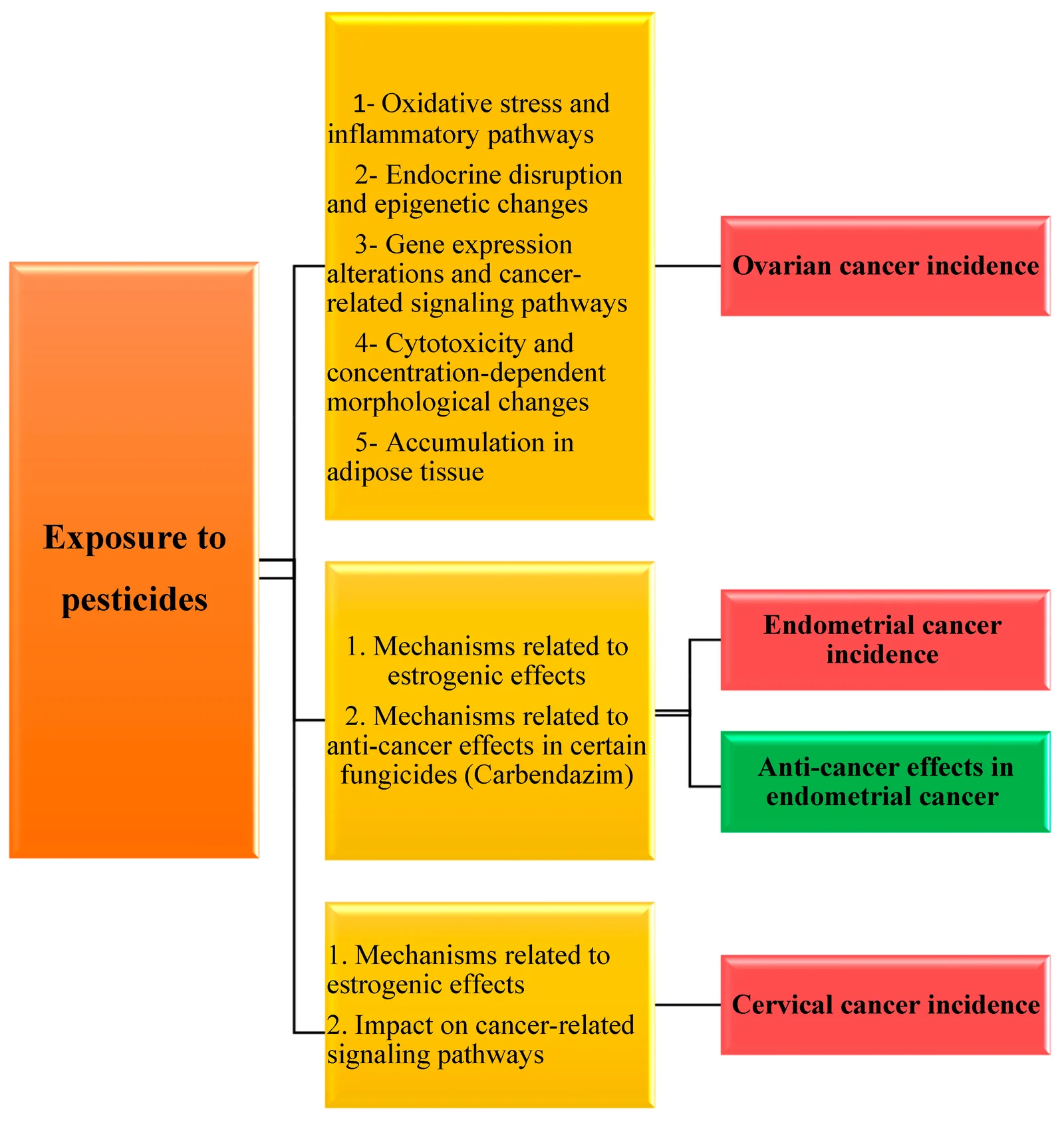
Summary of mechanisms involved in gynecological cancers.

Pesticides contribute to ovarian cancer through multiple mechanisms. Organochlorines such as β-HCH, DDE, and dieldrin increase reactive oxygen species (ROS) production. Exposure to these compounds upregulates pro-inflammatory cytokines (IL-6, TNF-α, IL-1β), leading to oxidative stress, inflammatory response activation, and DNA damage in ovarian epithelial cells. Additionally, the expression of inflammatory regulators like NF-κB and COX-2 is significantly increased [12] (Figure 1).

##### Endocrine Disruption and Epigenetic Changes

Pesticides including lindane, atrazine, endosulfan, dieldrin, methoxychlor (MXC), p,p′-DDT and their metabolites, p,p′-DDD and p,p′-DDE, chlorpyrifos, and fenitrothion disrupt endocrine function, contributing to ovarian cancer. Some pesticides like MXC possess estrogenic properties that stimulate ovarian cancer cell proliferation. Chlorpyrifos and MXC alter gonadotropin (FSH and LH) levels, stimulating pathways involved in ovarian cancer cell proliferation and invasion. For example, MXC induces hypermethylation of the estrogen receptor beta (ERβ) and increases DNA Methyltransferase 3 Alpha (*Dnmt3a*) genes, silencing tumor suppressor genes and contributing to ovarian cancer pathogenesis [22,43]. MXC also mimics estrogen, activating estrogen receptor-dependent pathways to promote BG-1 ovarian cancer cell growth [37].

##### Gene Expression Changes and Cancer-Related Signaling Pathways

MXC increases the expression of Cyclin D1 (a cell cycle promoter) and decreases Cyclin-Dependent Kinase Inhibitor 1A (*CDKN1A (p21)*) (a cell cycle inhibitor) [37]. Gene expression profiles in OCP-exposed ovarian cancer patients differed significantly from controls, with 146 genes upregulated and 13 downregulated. These genes were involved in cellular processes such as cytoskeleton reorganization via TGF-β, WNT, and Rho GTPases pathways, which play key roles in carcinogenesis. Twelve cancer-related genes (*NKIRAS1*, *TXNRD2*, *CCL23*, *IGFBP7*) were significantly correlated with high pesticide levels. Thus, pesticide exposure promotes ovarian cancer progression by altering gene expression related to cancer, signaling, and apoptosis [13]. Cytochrome P450 (*CYP*) family genes are one of the main enzymes in xenobiotic metabolism. *CYP* enzymes, by activating polycyclic aromatic hydrocarbons (PAHs), lead to the formation of reactive metabolites such as catechol estrogens and quinones and redox reactions such as o-quinones and diolepoxides, which damage DNA. Ultimately, they lead to mutations and the initiation of the carcinogenic process caused by exposure to organochlorine pesticides [44,45]. Furthermore, polymorphisms in *CYP* genes involved in steroid metabolism have been identified as genetic risk factors for hormone-related cancers by altering steroid hormone metabolism and modulating hormone activity [46]. Also, changes in steroid hormone metabolism due to the induction of certain *CYP* polymorphisms, by producing ROS, causing oxidative stress and changes in steroid hormone metabolism, are associated with hormone-dependent cancers [44]. In addition, several studies have shown that *CYP1A1*, *CYP1A2*, *CYP1B1*, *CYP3A4*, *CYP17A1*, and *CYP19* variants are associated with susceptibility to ovarian cancer [47]. In addition, some polymorphisms, by increasing the activation of procarcinogens and reducing detoxification, increase the risk of tumorigenesis in individuals with a genetic predisposition [44].

##### Cytotoxicity and Morphological Changes Dependent on Concentration

Exposure to OCPs, endosulfan, and heptachlor at concentrations >30 µM caused cytotoxicity and reduced survival of superficial ovarian epithelial cells. At <20 µM, no effect on survival was observed. At 10 µM, these pesticides induced morphological changes, including cellular aggregation and mass structures in HSOE cells. At this concentration, Cadherin 1(*CDH1*) gene and E-cadherin protein expression and pro-inflammatory cytokines (IL-6, IL-1β, TNF-α) were significantly increased [40].

##### Accumulation in Adipose Tissue

Pesticides accumulate in adipose tissue. Consequently, EOC patients with higher BMI exhibited greater pesticide accumulation [13].

#### 3.1.3. Role of Pesticides in Ovarian Cancer Progression

Organochlorine pesticides influence both the onset and progression of ovarian cancer. Laboratory studies on human granulosa cell tumors showed that exposure to p,p′-DDT, p,p′-DDE, and hexachlorobenzene (HCB) increases cell migration and disrupts lymphatic endothelial barriers, promoting ovarian cancer. These pesticides activate the insulin-like growth factor 1 receptor (*IGF1R*) signaling pathway and increase matrix metalloproteinase-2 (*MMP2*) activity, leading to extracellular matrix degradation and enhanced invasion/metastasis [39]. MXC significantly reduces the pro-apoptotic protein Bcl-2-associated X protein (*BAX*), increasing ovarian cancer cell survival [37].

### 3.2. Endometrial Cancer

#### 3.2.1. Association Between Pesticides and Endometrial Cancer Incidence

Endometrial cancer is one of the most common gynecological cancers worldwide, with 435,041 new cases and 91,640 deaths reported in 2019, representing a 132% and 63% increase in incidence and mortality, respectively, since 1990 [48]. Exposure to several classes of pesticides, including herbicides, insecticides, and fungicides, has been investigated as a potential risk factor for endometrial cancer. Jobs involving contact with these materials, such as livestock farming, agriculture, and poultry farming, show increased endometrial cancer rates [10] (Table 2).

##### Organochlorine Compounds

Concentrations of certain organochlorines, including p,p′-DDE, HCB, PCBs, and chlordanes, were higher in endometrial cancer patients than controls in Sweden, although the overall association between pesticide exposure and endometrial cancer was not statistically significant [27]. However, DDE levels were positively and significantly associated with endometrial cancer in white women in the USA [26]. The odds ratio for DDE was higher in postmenopausal women and those with advanced-stage (III and IV) disease [27].

##### Other Pesticides

Laboratory studies in mice showed that the insecticide Afidopyropen at concentrations of 1000 and 3000 ppm significantly increased uterine adenocarcinoma [28]. Similarly, a positive association was observed between exposure to the fungicide cyprodinil (CYP) and endometrial cancer [14].

#### 3.2.2. Mechanisms of Pesticide Action in Endometrial Cancer

##### Mechanisms Related to Estrogenic Effects

Hormones and estrogenic stimulation are common factors leading to endometrial cancer [23]. CYP may promote the metastatic potential of endometrial cancer cells to other tissues by upregulating metastasis-related proteins like cathepsin-D and MMP-9 via estrogen-dependent signaling pathways [14] (Figure 1).

##### Mechanisms Related to Anti-Cancer Effects

Carbendazim, a carbamate fungicide, binds to tubulin dimers’ β-tubulin subunits, preventing microtubule polymerization. This causes cell cycle arrest and cell death through microtubule instability and depolymerization. Carbendazim also exhibits potent anti-cancer properties. When used with nanotechnology and molecular targeting (aptamers), this toxic pesticide can be utilized in anti-cancer drug development [38] (Figure 1). 

### 3.3. Cervical Cancer

#### 3.3.1. Association Between Pesticides and Cervical Cancer Incidence

A study investigating the link between OCPs and reproductive system cancers found that cervical cancer had the highest prevalence. Mean concentrations of all measured pesticides and individual pesticides (α-HCH, β-HCH, γ-HCH, heptachlor, aldrin, p,p′-DDT, p,p′-DDD and p,p′-DDE) were significantly higher in the blood of women with reproductive cancers compared to controls [8]. Another study in Mexico measuring OCP levels in cervical cancer patients identified livestock farming areas as the most contaminated, followed by agricultural areas, with high levels of Endosulfan, Aldrin, 4,40-DDD, Heptachlor, α-HCH, β-HCH, γ-HCH, δ-HCH, Endrine Aldehyde, and Dieldrin [29]. In a Chinese study, exposure to Fluoro-phenoxy benzoic acid and dichlorovinyl-dimethyl propoxycarboxamide increased cervical cancer risk, though not significantly [31] (Table 2). 

#### 3.3.2. Mechanisms of Pesticide Action in Cervical Cancer

##### Mechanisms Related to Estrogenic Effects

In southwestern Punjab, extensive agricultural pesticide use has increased the risk of reproductive cancers, including cervical cancer. Organochlorines like endosulfan and heptachlor mimic estrogen, stimulating cancer cell proliferation. Fenvalerate and chlorpyrifos affect cell growth by activating estrogen receptors [16]. p,p′-DDT, acting as a xenoestrogen at concentrations of 20–120 nmol/L, suppressed the proliferation of cervical cancer (HeLa) cells [15]. Laboratory studies using HeLa-derived HELN ERα and HELN ERβ cell lines demonstrated that pesticides like o,p′-DDT, fenvalerate, and carbaryl interfere with estrogen signaling pathways, potentially causing cancer [41]. Xenoestrogens like p,p′-DDT and TCDD at 20–120 nmol/L also suppressed HeLa cell proliferation (Figure 1).

##### Impact on Cancer-Related Signaling Pathways

In cervical cancer patients exposed to 2,4-D, aspartate aminotransferase (AST) levels were significantly increased, correlating with high cancer risk [31]. p,p′-DDT suppressed BCL-2 (an anti-apoptotic protein) in HeLa cells and increased cyclin A and D protein levels [15]. Fenvalerate and chlorpyrifos increase angiogenesis and activate pathways involved in cell division and growth, such as PI3K and MAPK, contributing to carcinogenic risk [16].

#### 3.3.3. Role of Pesticides in Cervical Cancer Progression

When pesticides interact with HPV in the cervix, they increase the expression of oncogenes E6 and E7, enhancing the carcinogenic potential of cervical cells. Pesticides also induce pre-cancerous changes in cervical cells (CIN1 to CIN3), ultimately contributing to cervical cancer development [16].

**Table 1:** An overview of the included studies.

First Author (Year)	Country	Type of Study	Cancer Type of Interest	Pesticides Under Investigation
Cocco et al. (2000) [26]	Italy	Ecological (correlational) study using multivariate regression analysis on mortality data and DDE levels in adipose tissue samples from population samples of 22 U.S. states.	breast cancer uterine cancer (corpus uteri)	DDE (p,p′-dichlorodiphenyldichloroethylene, DDT (dichlorodiphenyltrichloroethane)
Koifman et al. (2002) [25]	Brazil	epidemiological ecologic study	ovarian cancer	pesticides
Hopenhayn-Rich et al. (2002) [33]	USA	Ecological Study	Ovarian Cancer	Atrazine
Hardell et al. (2004) [27]	Sweden	case–control study	endometrial cancer	polychlorinated biphenyls, hexachlorobenzene (HCB), p,p′-dichlorodiphenyl-dichloroethylene (p,p′-DDE), chlordanes, and polybrominated biphenyls
Tanaka et al. (2004) [36]	Japan	Experimental animal study/In vivo study	ovarian carcinogenesis	Atrazine
Lemaire et al. (2006) [41]	France	In Vitro Experimental Study	Cervical Cancer	DDT, Trans-Nonachlor, Chlordane, Fenvalerate, Toxaphene, Carbaryl, Pentachlorophenol, 2,4,5-T, Chlordecone/Kepone, Methoxychlor, Carbaryl, Endosulfan, Endrin, Dieldrin, Aldrin
Mathur et al. (2008) [8]	India	case–control study	reproductive tract cancers like cervical, uterine, vaginal and ovarian cancers	benzene hexa chlororide and its isomers, dieldrin, heptachlor, dichloro diphenyl trichloro ethane and its metabolites
Ndebele et al. (2010) [15]	USA	In vitro experimental study on HeLa cells	Cervical cancer	Coumestrol-a phytoestrogen, tetrachlorodioxin (TCDD)-a herbicide and DDT-a pesticide
Kim et al. (2014) [37]	Korea	In vitro experimental study	ovarian cancer	Methoxychlor and triclosan
Go et al. (2015) [22]	Korea	In vitro and in vivo experimental study	Ovarian cancer	Fenhexamid and cyprodinil
Zadnik et al. (2016) [23]	Slovenia	Review (Epidemiological trends)	Ovarian and endometrial cancers	Polychlorinated biphenyls, DDT, DDE
Inoue-Choi et al. (2016) [30]	USA	Prospective Cohort Study	ovarian cancer	Atrazine
Polanco Rodríguez Á et al. (2017) [29]	Mexico	Exploratory study	uterine cervix cancer	Organochlorine pesticides (Endosulfan I, Aldrin, 4,4′ DDD, δ-HCH, Dieldrin, DDE)
Kim et al. (2018) [14]	Korea	In vitro experimental study on Ishikawa cells	Endometrial cancer	Cyprodinil
Shah et al. (2018) [13]	India	Case–control study	Epithelial Ovarian Cancer	β-hexachlorocyclohexane (β-HCH), Heptachlor, Heptachlor epoxide B (HTEB), dichlorodiphenyldichloroethylene (p′p′-DDE), endosulfan-I
Van Cott et al. (2018) [28]	USA	Experimental animal study/In vivo study	Uterine adenocarcinoma	Afidopyropen
Sharma et al. (2019) [24]	India	case–control study	Epithelial ovarian cancer	Beta-hexachlorocyclohexane (β-HCH), endosulfan-I, endosulfan-II, dichlorodiphenyltrichloroethane (p′p′-DDT), dichlorodiphenyldichloroethylene (p′p′-DDE)
Tuna et al. (2019) [38]	Turkey	In vitro experimental study	Cervical cancer	Carbendazim
Shah et al. (2020) [12]	India	An in vitro study	Epithelial Ovarian Cancer	Organochlorine pesticides (OCPs) namely β-hexachlorocyclohexane (β-HCH), dichlorodiphenyldichloroethylene (DDE) and Dieldrin
Gogola-Mruk et al. (2021) [39]	Poland	In vitro experimental study	Ovarian granulosa cell tumor	Perfluorooctanoate, perfluorooctanesulfonate, 2,2-dichlorodiphenyldichloroethylene, polychlorinated biphenyl 153, and hexachlorobenzene
Panis et al. (2022) [34]	Brazil	ecological/observational study, using SISÁGUA report	Ovarian cancer	Alachlor, Aldrin-Dieldrin, Atrazine, Chlordane, DDT-DDD-DDE, Diuron, Glyphosate-AMPA, Lindane-γ-HCH, Mancozeb-ETU, Molinate, and Trifluralin
Shah et al. (2022) [40]	India	In vitro experimental study	epithelial ovarian cancer	Dichlorodiphenyldichloroethylene (DDE), Endosulfan, and Heptachlor
Krishnan et al. (2023) [16]	California	Review	Cervical Cancer, Ovarian Cancer	Heptachlor, Endosulfan, Fenvalerate, Chlorpyrifos
Renier et al. (2024) [35]	France	Cohort study	ovarian cancer	Triazine herbicide
Peñalver-Piñol et al. (2025) [10]	Spain	case–control study	Endometrial cancer	Pesticides, insecticides, fungicides, herbicides
Liu et al. (2025) [31]	China	Cross-sectional Study	Cervical Cancer	2,4-D, 4-fluoro-3-phenoxybenzoic acid, 3-phenoxybenzoic acid, Oxypyrimidine, Paranitrophenol, Dichlorovnl-dimeth prop carboacid
Wang et al. (2025) [49]	China	comprehensive Review include in vivo and in vitro study	Ovarian Cancer	DDT, DDE, MXC, HCB, Pyrethroids, Neonicotinoids, Carbamates, Atrazine, 2,4-dichlorophenoxyacetic acid, Acetochlor, Molinate, Paraquat, Fenoxaprop-ethyl, Diquat, Diuron, Trifluralin, Simazine, Mancozeb, Azoles, Vinclozolin, Chlorothalonil, Carbendazim, Fenarimol, Flutolanil, PCNB
Cooper et al. (2025) [32]	USA	Comprehensive Systematic Review	ovarian and uterine cancer	Atrazine

**Table 2:** Association of Different Pesticides with Ovarian, Endometrial, and Cervical Cancers.

Pesticide	Ovarian Cancer	Endometrial Cancer	Cervical Cancer	Study Type	References
α-BHC	✓		✓	Case–control/ in vitro study	[8,12]
β-BHC	✓		✓	Case–control/ in vitro study	[8,12]
γ-BHC (Lindane)	✓		✓	Case–control/ in vitro study/in vivo experimental study	[8,12,22]
Endosulfan	✓		✓	Case–control/In vitro and in vivo experimental study/Review/Exploratory study	[13,16,22,24,29,40]
Heptachlor	✓		✓	Case–control/ in vitro study/Review/Exploratory study	[8,12,13,16,24,29,40]
Methoxychlor	✓			Case–control/ in vitro study	[8,12,22,24,37]
Hexachlorobenzene (HCB)	✓	✓		Case–control/ in vitro study	[27,39]
Dieldrin	✓			Case–control/ in vitro study/in vivo experimental study/Exploratory study	[8,12,22,29]
Aldrin	✓		✓	Case–control	[8,29]
DDD	✓		✓	Case–control	[8,29]
DDE	✓	✓	✓	Case-control/ in vitro study/Ecological	[8,12,13,24,26,27,39]
DDT	✓		✓	Case–control/ in vitro study/in vivo experimental study	[8,12,15,22,,39,41]
Afidopyropen		✓		In vivo study (animal)	[28]
Fenitrothion	✓			In vitro and in vivo experimental study	[22]
Cyprodinil		✓		In vitro study/Review	[14,23]
Chlorpyrifos	✓		✓	In vitro and in vivo experimental study/Review	[16,22]

## 4. Discussion

This review examined the association between exposure to various pesticides, including fungicides, herbicides, and insecticides, and the incidence and progression of gynecological cancers. The analysis indicates that pesticides contribute to the development and progression of ovarian, endometrial, and cervical cancers through various mechanisms. Pesticides can impact ovarian cancer by producing ROS, increasing pro-inflammatory cytokines, causing DNA damage, disrupting endocrine function, and altering gene expression. Pesticides play a dual role in endometrial cancer; most contribute via estrogen mimicry, while some fungicides like carbendazim can halt cancer cell cycles and aid in treatment when used with nanotechnology. Similarly, pesticides cause cervical cancer and its progression through estrogenic effects and impacts on cancer-related signaling pathways. For cervical cancer, persistent HPV infection remains the main risk factor for cervical cancer. Therefore, the findings reviewed in this study should be interpreted as an environmental co-factor for cervical carcinogenesis, rather than an independent primary cause of cervical cancer.

Findings from reviewed studies suggest that pesticides can contribute to the onset and progression of reproductive cancers, including ovarian, endometrial, and cervical cancers [8,9,12,13,24,25,26,27,28,29]. A review indicated that pesticide exposure adversely affects the ovaries; women living in areas with widespread pesticide use have a higher risk of ovarian cancer compared to unexposed women [9]. Another study found a positive association between triazine pesticide exposure and ovarian cancer [40]. Similarly, exposure to certain organochlorines increased endometrial cancer risk [26], with higher pesticide concentrations found in diseased tissues as the disease stage advanced [27]. A longitudinal study showed that increased exposure to organochlorines was associated with a higher risk of reproductive cancers, particularly cervical cancer [16].

Although many studies reported a significant positive association between pesticide exposure and gynecological cancers, others found no significant link [24,27,30,31]. A review found no convincing association between atrazine exposure and reproductive cancers, including ovarian and uterine cancers [32]. Similarly, a longitudinal study found no significant association between drinking water atrazine levels and ovarian cancer [30,34]. Another study even suggested a protective effect of atrazine in drinking water against ovarian cancer [33]. Epidemiological studies have been conducted in various countries—including India, the United States, France, Sweden, Mexico, China, Brazil, Italy, South Korea, Slovenia, Turkey, Poland, Spain, and California—revealing diversity in the types and quantities of pesticides used across different geographic regions; these variations may contribute to the conflicting results observed such as the lack of a significant association for triazines and atrazine in the USA and France [32,33,35], contrasted with a positive association for organochlorines in India and Mexico [8,12,13,24,29]. It should be noted that some studies have shown a possible association between atrazine herbicides and an increased risk of breast cancer [50]. Therefore, the lack of a positive association with ovarian cancer in the reviewed studies should be interpreted with caution. These contradictions may stem from differences in exposure assessment methods (biomonitoring vs. environmental modeling), latency periods, and the presence of co-exposures such as nitrates and trihalomethanes in water sources. Consistent with this, no significant positive association was observed between organochlorines and endometrial cancer [27], and some studies found no link between specific pesticides and cervical cancer [31].

This narrative review focuses on the association between pesticide exposure and ovarian, endometrial, and cervical cancers. It integrates epidemiological results with experimental and laboratory evidence to understand effective mechanisms in gynecological cancer pathogenesis. The study also highlights the dual role of certain pesticides like carbendazim, which is carcinogenic but has anti-cancer properties under specific conditions. However, limitations include the inability to establish causality in observational research and contradictory findings. Further longitudinal studies with diverse pesticides are needed.

The findings emphasize the importance of minimizing occupational and residential exposure to pesticides among women, especially women who work in agriculture or live in areas with heavy pesticide use. They highlight the need for more effective screening for the health of women employed in or exposed to pesticide environments. Educating at-risk women on PPE is essential. Understanding pesticide mechanisms in gynecological cancers allows for the identification of new biomarkers for early detection and prevention. Additionally, the dual role of pesticides like carbendazim offers possibilities for their use in anti-cancer drug therapy.

## 5. Conclusions

This narrative review demonstrates that pesticide exposure, particularly to organochlorine compounds, plays a significant role in the onset and progression of gynecological cancers. Various mechanisms have been identified, including oxidative stress, altered gene expression and cancer-related signaling pathways, endocrine disruption, estrogen mimicry, and changes in pro-inflammatory factors. The dual carcinogenic and anti-cancer effects of certain pesticides have also been demonstrated. Finally, due to contradictory findings regarding the causal relationship between pesticide exposure and reproductive cancers, further studies are required.

## Data Availability

No new data were created or analyzed in this study. Data sharing is not applicable to this article.

## References

[1] CDC CfDCaP Gynecologic Cancers 2024. https://www.cdc.gov/gynecologic-cancer/about/index.html.

[2] Bray F., Laversanne M., Sung H., Ferlay J., Siegel R.L., Soerjomataram I., Jemal A. (2024). Global cancer statistics 2022: GLOBOCAN estimates of incidence and mortality worldwide for 36 cancers in 185 countries. CA Cancer J. Clin..

[3] Jouya S., Shahabinia Z., Mazidimoradi A., Allahqoli L., Salehiniya H., Lee D.Y. (2026). Cervical Cancer Epidemiology: Global Incidence, Mortality, Survival, Risk Factors, and Equity in HPV Screening and Vaccination. J. Clin. Med..

[4] Crosbie E.J., Kitson S.J., McAlpine J.N., Mukhopadhyay A., Powell M.E., Singh N. (2022). Endometrial cancer. Lancet.

[5] Webb P.M., Jordan S.J. (2024). Global epidemiology of epithelial ovarian cancer. Nat. Rev. Clin. Oncol..

[6] Mazidimoradi A., Momenimovahed Z., Allahqoli L., Tiznobaik A., Hajinasab N., Salehiniya H., Alkatout I. (2022). The global, regional and national epidemiology, incidence, mortality, and burden of ovarian cancer. Health Sci. Rep..

[7] Momenimovahed Z., Tiznobaik A., Taheri S., Salehiniya H. (2019). Ovarian cancer in the world: Epidemiology and risk factors. Int. J. Women’s Health.

[8] Mathur V., John P.J., Soni I., Bhatnagar P. (2008). Blood levels of organochlorine pesticide residues and risk of reproductive tract cancer among women from Jaipur, India. Adv. Exp. Med. Biol..

[9] Wang L., Ma X., Liu J. (2025). Adverse Effects of Pesticides on the Ovary: Evidence from Epidemiological and Toxicological Studies. Environ. Health.

[10] Peñalver-Piñol A., Benavente Y., Frias-Gomez J., Alguacil J., Santibañez M., Contreras-Llanes M., Peremiquel-Trillas P., López-Querol M., Paytubi S., Pelegrina B. (2023). Occupational exposure to pesticides and endometrial cancer in the Screenwide case-control study. Environ. Health.

[11] Alhamlan F.S., Alfageeh M.B., Al Mushait M.A., Al-Badawi I.A., Al-Ahdal M.N. (2021). Human Papillomavirus-Associated Cancers. Advances in Experimental Medicine and Biology.

[12] Shah H.K., Sharma T., Banerjee B.D. (2020). Organochlorine pesticides induce inflammation, ROS production, and DNA damage in human epithelial ovary cells: An in vitro study. Chemosphere.

[13] Shah H.K., Bhat M.A., Sharma T., Banerjee B.D., Guleria K. (2018). Delineating Potential Transcriptomic Association with Organochlorine Pesticides in the Etiology of Epithelial Ovarian Cancer. Open Biochem. J..

[14] Kim B.G., Kim J.W., Kim S.M., Go R.E., Hwang K.A., Choi K.C. (2018). 3,3′-Diindolylmethane Suppressed Cyprodinil-Induced Epithelial-Mesenchymal Transition and Metastatic-Related Behaviors of Human Endometrial Ishikawa Cells via an Estrogen Receptor-Dependent Pathway. Int. J. Mol. Sci..

[15] Ndebele K., Graham B., Tchounwou P.B. (2010). Estrogenic Activity of Coumestrol, DDT, and TCDD in Human Cervical Cancer Cells. Int. J. Environ. Res. Public Health.

[16] Krishnan K., Yen A., Bains N. Chemical Analysis of Groundwater in Southwest Punjab: The Use of Pesticides and Its Link to Cervical Cancer. https://www.researchgate.net/publication/370632470_Chemical_Analysis_of_Groundwater_in_Southwest_Punjab_The_Use_of_Pesticides_and_its_Link_to_Cervical_Cancer.

[17] National Institute of Environmental Health Sciences (2025). Pesticides. https://www.niehs.nih.gov/health/topics/agents/pesticides.

[18] World Health Organization (2020). Chemical Safety: Pesticides. https://www.who.int/news-room/questions-and-answers/item/chemical-safety-pesticides.

[19] Damalas C.A., Eleftherohorinos I.G. (2011). Pesticide exposure, safety issues, and risk assessment indicators. Int. J. Environ. Res. Public Health.

[20] Davis J.R., Brownson R.C., Garcia R. (1992). Family pesticide use in the home, garden, orchard, and yard. Arch. Environ. Contam. Toxicol..

[21] Jaga K., Dharmani C. (2003). Sources of exposure to and public health implications of organophosphate pesticides. Rev. Panam. Salud Publica.

[22] Go R.E., Kim C.W., Choi K.C. (2015). Effect of fenhexamid and cyprodinil on the expression of cell cycle- and metastasis-related genes via an estrogen receptor-dependent pathway in cellular and xenografted ovarian cancer models. Toxicol. Appl. Pharmacol..

[23] Zadnik V., Krajc M. (2016). Epidemiological trends of hormone-related cancers in Slovenia. Arh. Hig. Rada Toksikol..

[24] Sharma T., Banerjee B.D., Thakur G.K., Guleria K., Mazumdar D. (2019). Polymorphism of xenobiotic metabolizing gene and susceptibility of epithelial ovarian cancer with reference to organochlorine pesticides exposure. Exp. Biol. Med..

[25] Koifman S., Koifman R.J., Meyer A. (2002). Human reproductive system disturbances and pesticide exposure in Brazil. Cad. Saude Publica.

[26] Cocco P., Kazerouni N., Zahm S.H. (2000). Cancer mortality and environmental exposure to DDE in the United States. Environ. Health Perspect..

[27] Hardell L., van Bavel B., Lindström G., Björnfoth H., Orgum P., Carlberg M., Sörensen C.S., Graflund M. (2004). Adipose tissue concentrations of p,p′-DDE and the risk for endometrial cancer. Gynecol. Oncol..

[28] Van Cott A., Frericks M., Hastings C., Honarvar N., Flick B., Fabian E., van Ravenzwaay B. (2018). Uterine adenocarcinoma in the rat induced by afidopyropen. An analysis of the lesion’s induction, progression and its relevance to humans. Regul. Toxicol. Pharmacol..

[29] Polanco Rodríguez Á.G., Riba López M.I., DelValls Casillas T., Araujo León J.A., Mahjoub O., Prusty A.K. (2017). Monitoring of organochlorine pesticides in blood of women with uterine cervix cancer. Environ. Pollut..

[30] Inoue-Choi M., Weyer P.J., Jones R.R., Booth B.J., Cantor K.P., Robien K., Ward M.H. (2016). Atrazine in public water supplies and risk of ovarian cancer among postmenopausal women in the Iowa Women’s Health Study. Occup. Environ. Med..

[31] Liu Y., Li K., Li C., Feng Z., Cai Y., Zhang Y., Hu Y., Wei X., Yao P., Liu X. (2024). Pesticides, cancer, and oxidative stress: An application of machine learning to NHANES data. Environ. Sci. Eur..

[32] Cooper R.L., Simpkins J.W., Breckenridge C. (2025). Effects of atrazine on the HPG and HPA axes and steroidogenic pathways in females: Relevance to reproductive function and breast, ovarian and uterine cancer. Front. Toxicol..

[33] Hopenhayn-Rich C., Stump M.L., Browning S.R. (2002). Regional assessment of atrazine exposure and incidence of breast and ovarian cancers in Kentucky. Arch. Environ. Contam. Toxicol..

[34] Panis C., Candiotto L.Z.P., Gaboardi S.C., Gurzenda S., Cruz J., Castro M., Lemos B. (2022). Widespread pesticide contamination of drinking water and impact on cancer risk in Brazil. Environ. Int..

[35] Renier M., Hippert J., Louis-Bastien W., Tual S., Meryet-Figuiere M., Vigneron N., Marcotullio E., Baldi I., Lebailly P. (2024). Agricultural exposure and risk of ovarian cancer in the AGRIculture and CANcer (AGRICAN) cohort. Occup. Environ. Med..

[36] Tanaka T., Kohno H., Suzuki R., Sugie S. (2004). Lack of modifying effects of an estrogenic compound atrazine on 7,12-dimethylbenz(a)anthracene-induced ovarian carcinogenesis in rats. Cancer Lett..

[37] Kim J.Y., Yi B.R., Go R.E., Hwang K.A., Nam K.H., Choi K.C. (2014). Methoxychlor and triclosan stimulates ovarian cancer growth by regulating cell cycle- and apoptosis-related genes via an estrogen receptor-dependent pathway. Environ. Toxicol. Pharmacol..

[38] Tuna B.G., Atalay P.B., Kuku G., Acar E.E., Kara H.K., Yilmaz M.D., Ozalp V.C. (2019). Enhanced antitumor activity of carbendazim on HeLa cervical cancer cells by aptamer mediated controlled release. RSC Adv..

[39] Gogola-Mruk J., Hoffmann-Młodzianowska M., Kamińska K., Ptak A. (2021). Mixtures of persistent organic pollutants increase ovarian granulosa tumor cell line migration and spheroid invasion by upregulating MMP2 expression and activity via IGF1R. Toxicology.

[40] Shah H.K., Banerjee B.D., Thakur G.K., Guleria K. (2022). Organochlorine pesticides induce epithelial as well as inflammatory mediators following exposure to human ovarian surface epithelial cells: An in vitro study. J. Biochem. Mol. Toxicol..

[41] Lemaire G., Mnif W., Mauvais P., Balaguer P., Rahmani R. (2006). Activation of α-and β-estrogen receptors by persistent pesticides in reporter cell lines. Life Sci..

[42] Siegel R.L., Kratzer T.B., Giaquinto A.N., Sung H., Jemal A. (2025). Cancer statistics, 2025. CA Cancer J. Clin..

[43] Zama A.M., Uzumcu M. (2009). Fetal and neonatal exposure to the endocrine disruptor methoxychlor causes epigenetic alterations in adult ovarian genes. Endocrinology.

[44] Docea A.O., Vassilopoulou L., Fragou D., Arsene A.L., Fenga C., Kovatsi L., Petrakis D., Rakitskii V.N., Nosyrev A.E., Izotov B.N. (2017). CYP polymorphisms and pathological conditions related to chronic exposure to organochlorine pesticides. Toxicol. Rep..

[45] Elfaki I., Mir R., Almutairi F.M., Duhier F.M.A. (2018). Cytochrome P450: Polymorphisms and Roles in Cancer, Diabetes and Atherosclerosis. Asian Pac. J. Cancer Prev..

[46] Friedberg T. (2001). Cytochrome P450 polymorphisms as risk factors for steroid hormone-related cancers. Am. J. Pharmacogenomics.

[47] Al-Saraireh Y.M., Alshammari F., Abu-Azzam O.H., Al-Dalain S.M., Al-Sarayra Y.M., Haddad M., Makeen H., Al-Qtaitat A., Almermesh M., Al-Sarayreh S.A. (2023). Targeting Cytochrome P450 Enzymes in Ovarian Cancers: New Approaches to Tumor-Selective Intervention. Biomedicines.

[48] Yang L., Yuan Y., Zhu R., Zhang X. (2023). Time trend of global uterine cancer burden: An age-period-cohort analysis from 1990 to 2019 and predictions in a 25-year period. BMC Women’s Health.

[49] Wang Z., Fu Y., Cai Q., Zong L. (2026). Deciphering pathogenic mechanisms of BDCPP exposure in endometrial cancer progression via an integrated approach combining network toxicology, machine learning, and molecular docking. Int. J. Surg..

[50] Jiang H., Liu Y., Wang C., Song Y., Wu F., Yin Y., Yang Z. (2025). Nanoparticles Loaded with Pralidoxime Wrapped in Tumor Cell Membranes: A New Strategy to Counteract the Central Nervous System Effects of Organophosphate Poisoning. Int. J. Nanomed..

